# Unveiling Lipidomic Alterations in Metabolic Syndrome: A Study of Plasma, Liver, and Adipose Tissues in a Dietary-Induced Rat Model

**DOI:** 10.3390/nu16203466

**Published:** 2024-10-13

**Authors:** Snjezana Petrovic, Thomai Mouskeftara, Marija Paunovic, Olga Deda, Vesna Vucic, Maja Milosevic, Helen Gika

**Affiliations:** 1 Group for Nutritional Biochemistry and Dietology, Centre of Research Excellence in Nutrition and Metabolism, Institute for Medical Research, National Institute of Republic of Serbia, University of Belgrade, 11000 Belgrade, Serbia; marija.paunovic@imi.bg.ac.rs (M.P.); vesna.vucic@imi.bg.ac.rs (V.V.); 2 Laboratory of Forensic Medicine & Toxicology, Department of Medicine, Aristotle University of Thessaloniki, 54124 Thessaloniki, Greece; 3 Biomic AUTh, Center for Interdisciplinary Research and Innovation (CIRI-AUTH), Balkan Center B1.4, 10th km Thessaloniki-Thermi Rd, P.O. Box 8318, 57001 Thessaloniki, Greece; 4 Group for Neuroendocrinology, Institute for Medical Research, National Institute of Republic of Serbia, University of Belgrade, 11000 Belgrade, Serbia; mmilosevic@imi.bg.ac.rs

**Keywords:** untargeted lipidomics, metabolic syndrome, plasma, liver, adipose tissue, animal model

## Abstract

Metabolic syndrome (MetS) is a complex condition characterized by fat accumulation, dyslipidemia, impaired glucose control and hypertension. In this study, rats were fed a high-fat high-fructose (HFF) diet in order to develop MetS. After ten weeks, the dietary-induced MetS was confirmed by higher body fat percentage, lower HDL-cholesterol and increased blood pressure in the HFF-fed rats compared to the normal-fed control animals. However, the effect of MetS development on the lipidomic signature of the dietary-challenged rats remains to be investigated. To reveal the contribution of specific lipids to the development of MetS, the lipid profiling of rat tissues particularly susceptible to MetS was performed using untargeted UHPLC-QTOF-MS/MS lipidomic analysis. A total of 37 lipid species (mainly phospholipids, triglycerides, sphingolipids, cholesterol esters, and diglycerides) in plasma, 43 lipid species in liver, and 11 lipid species in adipose tissue were identified as dysregulated between the control and MetS groups. Changes in the lipid signature of selected tissues additionally revealed systemic changes in the dietary-induced rat model of MetS.

## 1. Introduction

Metabolic syndrome (MetS) rose to be one of the biggest health issues facing modern humanity [1,2]. The interaction of associated obesity, dyslipidemia, hypertension, and hyperglycemia forms the basis for serious health threats such as cardiovascular diseases (CVD), non-alcoholic fatty liver disease (NAFLD), and type 2 diabetes mellitus (T2DM) [3]. In addition to numerous studies in humans, various animal models have been used to investigate the biochemical and molecular pathways of MetS development. Among them, dietary-induced rat models of MetS proved to be particularly suitable due to their similarity with human biological and (phato)physiological features [4]. In our previous study, we reported the appearance of MetS characteristics in Wistar rats treated with a customised high-fat high-fructose (HFF) diet for 10 weeks [5].

Lipids are one of the main components of the diet. They primarily serve as a sources of energy but are also the building blocks of cell and tissue structures and serve as a means of transport for fat-soluble nutrients [6]. Although lipid layers are necessary for maintaining thermoregulation because they protect vital organs and insult neurons, excessive accumulation of lipids in the body, as in obesity and MetS, represents a significant health risk. There is large body of papers reporting an association between feeding obesogenic diet in experimental animals, increased lipid deposition, and disturbed tissue composition and function [7]. Our already published data showed a significantly higher proportion of adipose tissue, lower plasma HDL-cholesterol (HDL-c) level, impaired glucose tolerance, increased systolic blood pressure, impaired histomorphology in liver, adipose and pancreatic tissues, and intensified oxidative stress burden, inflammation, and lipid deposition in the liver of dietary-challenged rats, all of which together confirm the occurrence of MetS in our HFF-fed rats [5,8].

Lipidomic studies on rat models provide critical insights into the mechanisms underlying MetS, emphasizing the importance of lipid metabolism in MetS development and progression. Recent studies using up-to-date lipidomic analysis have found significant differences in the occurrence of lipid species and their ratios in the tissues of animals fed hypercaloric diets compared to animals fed normal diets. Guirro et al. reported increased levels of diacylglycerols and phosphatidylcholines in rats fed an obesogenic diet, while Jin et al. found that triglyceride (TG) accumulation in non-adipose tissues serves as a reflection of lipotoxicity, a condition often observed in MetS [9,10]. Altered phospholipid composition in plasma and tissues, especially phosphatidylcholine and phosphatidylethanolamine levels, indicates disrupted membrane integrity and cellular signalling pathways [11]. These changes, together with elevated ceramide levels, and dysregulated balance between saturated and unsaturated fatty acids can exacerbate insulin resistance and promote inflammatory responses in affected tissues [12,13,14]. A comprehensive analysis revealed dysregulation of specific lipid classes, including glycerolipids, glycerophospholipids, sphingolipids, and fatty acids in the liver, and TG in the visceral adipose tissue of high-fat-diet-fed rats compared to normal-fed controls [15]. Additionally, oxylipins, which are bioactive lipid mediators derived from polyunsaturated fatty acids, have been shown to be altered in rat models of MetS [16].

In this study, we focused on gaining deeper insight into the HFF-diet-induced changes in the lipid signatures of three tissues particularly susceptible to MetS: plasma, liver, and adipose tissue. In particular, the study strives to reveal lipids that display significant changes in all three matrices and/or that potentially could be assumed to be biomarkers of MetS. Untargeted lipidomics analysis using liquid chromatography coupled to mass spectrometry (LC-MS) was performed to reveal the effects of the HFF diet on lipid profiles in a rat model of MetS in order to contribute to better understanding of physiological pathways involved in the onset and development of MetS.

## 2. Materials and Methods

### 2.1. Chemicals and Materials

Methanol (MeOH), acetonitrile (ACN), methyl-tert-butyl-ether (MTBE; ≥99%), chloroform (CHCl_3_), and formic acid (all ULC/MS-CC/SFC grade) were obtained from CHEM-LAB NV (Zedelgem, Belgium). Isopropanol (IPA) was purchased from Fisher Scientific (International Inc., Hampton, NH, USA). Ammonium formate (NH_4_HCO_2_; MS grade) was obtained from Sigma-Aldrich (Merck, Darmstadt, Germany). Deionized water (ddH_2_O) was ultrapurified using a Millipore (Bedford, MA, USA) instrument delivering water quality with a resistivity of ≥18.2 MΩ∙cm.

### 2.2. Animal Study

The experiments were carried out on 4-month-old male Wistar rats, which were randomly divided into two groups (*n* = 6 each). Our decision to use only six rats per group stemmed from the principles of the 3Rs (replacement, reduction, and refinement) and animal welfare [17]. The control group was placed on a standard chow diet (Agrofirm, Pozarevac, Serbia) and drank tap water; the HFF group was placed on a HFF diet of standard rat chow enriched with 25% sunflower oil, 20% fructose, and 0.1% cholic acid (HFF and water). The animals were kept under controlled conditions, a 12 h light–dark cycle, and 22 ± 2 °C and had free access to food and liquid (ad libitum). All experimental procedures were carried out according to the National Law of Animal Welfare (“Official Gazette of RS” 41/09 and 39/10) and the Directive 2010/63/EU. The study protocol was approved by the Ethics Committee of the Institute for Medical Research, National Institute of Republic of Serbia, University of Belgrade, Serbia, and Veterinary Administration, Ministry of Agriculture, Forestry and Water Management, Republic of Serbia (No. 323-07-06069/2019- 05), 26 June 2019, and in line with the ARRIVE protocol.

### 2.3. Physiological and Biochemical Measurements

For the assessment of food and liquid consumption, body mass, and adiposity, Intake of food and liquid was measured once per week and expressed as average intake throughout the study. Body mass was also measured weekly and presented as the baseline mass before the treatment and the final mass at the end of nutritive intervention. After the animals were sacrificed (4% isoflurane anesthesia was applied), total visceral fat was collected, and the obtained mass was used to calculate the percentage of final body mass. Blood samples were collected as well and centrifuged at 3000× *g* and 4 °C for 15 min to separate plasma. The levels of TG, total cholesterol (TC), HDL-cholesterol (HDL-c), and LDL-cholesterol (LDL-c) were determined using a clinical chemistry analyzer (Cobas c111, Roche Diagnostics, Basel, Switzerland) and Roche Diagnostics kits. Glucose levels were detected in the blood obtained from the tail tip using a glucometer (Contour Plus, Bayer, Germany). Blood pressure measurement was performed by recording the systolic blood pressure in conscious animals using the non-invasive tail–cuff method in animals previously trained for adaptation to the method (Rat Tail Cuff Method Blood Pressure Systems (MRBP-R), IITC Life Science Inc., Saint Petersburg, FL, USA). At least four measurements were recorded to obtain a mean result [5].

### 2.4. Sample Preparation for Lipid Analysis

For LC-QTOF-MS lipidomics analysis of plasma, 50 µL of the samples were thawed on ice for 30 min. The lipid extraction was performed by adding 375 µL MeOH and 1250 µL of MTBE in the samples, followed by vigorous vortexing and shaking for 30 min at ambient temperature. Phase separation was enhanced by adding 375 µL of H_2_O, and the samples were shaken for another 10 min at ambient temperature. After the incubation period ended, samples were centrifuged for 10 min at 4 C and 11.180 g. The organic phase was collected and transferred into 2 mL Eppendorf tubes, resulting in two different aliquots (one for positive and one for negative mode), before being evaporated to dryness in vacuo (SpeedVac, Eppendorf Austria GmbH, Wien, Austria). The dried samples were reconstituted with IPA (200 μL for negative ionization mode and 400 μL for positive ionization mode).

For lipid extraction from liver tissues, the samples were thawed on ice for 1 h, weighed and transferred to 2.0 mL Eppendorf tubes containing 1.0 mm ceramic beads. The organic solvent mixture of MTBE-MeOH 3:1 (*v*/*v*) was added to the tissue, proportionally to weight and up to 1200 μL for the maximum weight. The tissues were homogenized using a bead mill homogenizer (BEAD RUPTOR ELITE, Omni International, Kennesaw, Georgia), through 4 cycles of a 30 s duration and with speed set at 6.00 m/s. After homogenization, the mixtures were centrifuged at 4 °C for 30 min at 16.770 g, and two replicates of the supernatant, 300 µL each, were transferred to a 1.5 mL Eppendorf tube, followed by evaporation to dryness in vacuo (SpeedVac, Eppendorf Austria GmbH, Wien, Austria). The dried samples were reconstituted in 1000 μL of IPA for analysis in positive and negative ionization modes. Detailed information about the exact extraction solvent volumes and the extraction procedure is provided in Table S1. A modified Folch protocol was employed for the extraction of lipids from visceral adipose tissue. The tissue was weighed and transferred to a 2.0 mL Eppendorf tube containing 1.0 mm ceramic beads. The volume of the extraction solvents was adjusted proportionally based on the weight of the tissue, as outlined in Table S1. Specifically, in 10 mg of adipose tissue, 200 µL of MeOH was added, and the tissue was homogenized for 4 cycles of 30 s each at a speed of 6.00 m/s. The homogenates were transferred to new Eppendorf tubes, and subsequently, 640 µL of a CHCl_3_-MeOH 7:1 (*v*/*v*) was added, followed by 30 min of vortexing at ambient temperature. Afterward, 360 μL of CHCl_3_ was added and vortexed for an additional 30 min. To enhance phase separation, 180 μL of H_2_O was added, and the samples were centrifuged for 30 min at 16.770× *g* and 4 °C. Two replicates of the lower phase, each containing 200 µL, were collected in new 1.5 mL Eppendorf tubes. The dry residues were reconstituted in 500 μL of IPA and diluted 40 times with the same solvent.

A pooled sample (Quality Control, QC) was prepared by mixing equal volumes of each supernatant for each matrix to ensure the quality control of the analysis. Group-specific QC samples were prepared for each of the two studied groups, i.e., the controls and HFF-fed rats, by mixing equal volumes of the supernatants from the samples of the same group and matrix. Diluted QCs (1:2, 1:4, 1:6, 1:8) were also prepared for the evaluation of the dilution integrity of the detected lipids.

Each matrix was analyzed separately in a different analytical batch. All samples were analyzed in a randomized order, with QC samples being analyzed every 5 individual samples, resulting in a total of 6 QC samples being analyzed. Initially, blank samples, procedural blank samples, 4 QCs for column equilibration, and diluted QCs were analyzed, followed by group-specific QCs and individual samples.

### 2.5. Analytical Instrumentation and Conditions

A UHPLC Elute system equipped with an Elute autosampler was used. Lipids were separated on an Acquity UPLC CSH C18, 2.1 × 100 mm, 1.7 μm column (Waters Ltd., Elstree, UK) equipped with a pre-column Acquity UPLC CSH C18Van-Guard (Waters Ltd., Elstree, UK). The mobile phase A consisted of ACN/H_2_O 60:40 (*v*/*v*), 10 mM ammonium formate, and 0.1% formic acid, and the mobile phase B consisted of IPA/ACN 90:10 (*v*/*v*) and 0.1% formic acid. The gradient was as follows: 60–57% A (0.0–2.0 min), 57–50% A (2.0–2.1 min), 50–46% A (2.1–12.0 min), 46–30% A (12.0–12.1 min), 30–1% A (12.1–18 min), 1–60% A (18.0–18.1 min) and 60% A (18.1–20.0 min), with a total analysis time of 20 min. The flow rate was set at 0.4 mL/min and the column maintained at 55 °C. The autosampler vial tray was maintained at 8 °C, and the needle was initially washed with 1000 μL of the strong wash solvent IPA/ACN 90:10 (*v*/*v*), followed by 1000 μL of the weak wash solvent ACN/H_2_O 60/40 (*v*/*v*) before and after each injection. The injection volume was adjusted for the 3 different matrices and ionization modes. For the analysis of plasma and liver samples, 5 μL was injected in positive mode, while for negative mode, the injection volume was 8 μL. Adipose tissue samples were analyzed only in positive mode, and the injection volume was set at 5 μL.

A TIMS TOF mass spectrometer (Bruker, Bremen, Germany) was used in both positive and negative ionization mode for MS data acquisition, performing data-dependent acquisition (DDA) for MS/MS analyses. The settings in ESI were as follows: capillary voltage of ±4.2 kV, dry temperature of 200 °C, dry gas 10 L/min and nebulizer gas 2 Bar. Auto MS/MS was applied using dynamic MS/MS spectra acquisition with 6 and 10 Hz as minimum and maximum spectra rates, respectively. In DDA analysis, MS/MS spectra were selected for the 10 most intense ions per scan. The collision energy was set at 20 V for precursor ions below 100 *m*/*z*, 30 V for precursor ions with *m*/*z* ranging from 100 to 1000, and 40 V for precursor ions with *m*/*z* ranging from 1000 to 2000 *m*/*z*. Calibrant (sodium formate, 10 mM) was infused into MS at a 10 µL/h flow rate in the first 0.2 min of each analysis.

### 2.6. Data Analysis and Visualization

The acquired raw spectra from TIMS-TOF were recalibrated with sodium formate clusters using Data Analysis software (version 5.3, Bruker Bremen, Germany) and converted to mzML by MSConvert (ProteoWizard 3.0.11567). Retention time alignment and feature grouping were performed prior to chromatographic peak detection using an in-house XCMS script in R programming (version 3.2.0). Lipids containing missing values >20% in all samples and those with QC CV values >30% were excluded from the statistical analysis. Raw data were normalized using QC samples [18]. Principal component analysis (PCA), partial and orthogonal–partial least-squares discriminant analysis (PLS, OPLS-DA) were performed using the SIMCA 13.0.3 (UMETRICS AB, Umea, Sweden) software [19]. The filter S-plot was used, applying absolute *p* and *p* (corr) cutoff values of >|0.05| and |0.5|, respectively, for identifying significant features. The quality of the constructed models (such as goodness-of-fit in the X (R^2^X) and Y (R^2^Y) variables, predictability (Q^2^YCV), and *p* values from CV ANOVA analysis) were provided and calculated by the software. Logarithmic transformation of the data and Pareto scaling were used in all models in plasma and liver tissue, while only Pareto scaling was used in adipose tissue. Univariate statistical analysis was performed in GraphPad Prism v8.0.1 software to assess the differences between the study groups. Normal distribution was evaluated by the Shapiro–Wilk test. A Student’s test was conducted for normally distributed lipids, while the Mann–Whitney U test was used for non-normally distributed lipids. The Chi-square test was utilized in categorical parameters. Additionally, only lipids that were found to be significant in both multivariate and univariate statistical analysis were considered statistically significant.

### 2.7. Identification of the Statistically Significant Lipids

The statistically significant lipid species were identified by Lipostar2 (version 2.0.2, Molecular Discovery Ltd., Hertfordshire, UK) equipped with the LIPID MAPS structure database (version September 2021) [20]. The raw files were imported directly and aligned using the default settings. The algorithm Savitzky-Golay was used for automatic peak picking with the following parameters: window size set to 7, degree to 2, multi-pass iterations to 1, and a minimum S/N ratio of 3. Mass tolerance settings were set to 10 ppm with an RT tolerance of 0.2 min. Filters designed to retain lipids with isotopic patterns and retain lipids with MS/MS were applied as well. The following parameters were used for lipid identification: 5 ppm precursor ion mass tolerance and 20 ppm product ion mass tolerance. The automatic approval was performed to keep structures with a quality of 3–4 stars. More particularly, glycerolipids (cholesteryl esters, diglycerides, and triglycerides) were identified as [M + NH4]^+^ adducts in positive mode. Sphingolipids (ceramides, sphingomyelins) were identified as [M + HCOO]^−^ or [M + Cl]^−^ adducts in negative mode and in their protonated form [M + H]^+^ in positive mode. Glycerophospholipids such as PC were found as [M + HCOO]^−^ and [M + H]^+^ adducts in negative and positive mode, respectively, while PE species were identified as [M−H]^−^.

## 3. Results

### 3.1. Animal Model—Primary Findings

After 10 weeks of nutritive intervention, significant differences between experimental groups were detected (Table 1), given that HFF rats had an increased percentage of body fat (6.58 ± 1.21 vs. 3.24 ± 0.44, *p* < 0.001), a decreased level of HDL-c (0.80 ± 0.14 vs. 0.98 ± 0.18, *p* < 0.05), and elevated systolic blood pressure (148 ± 7 vs. 126 ± 9 mm Hg, *p* < 0.05), which were parameters corresponding to the onset and development of MetS in our rat model, as previously described in our recently published paper [5]. However, no differences in final body mass and levels of total cholesterol, TG, and glucose were found between the HFF and control group.

### 3.2. Lipidomic Profiling in Plasma Samples

**Figure 1:**
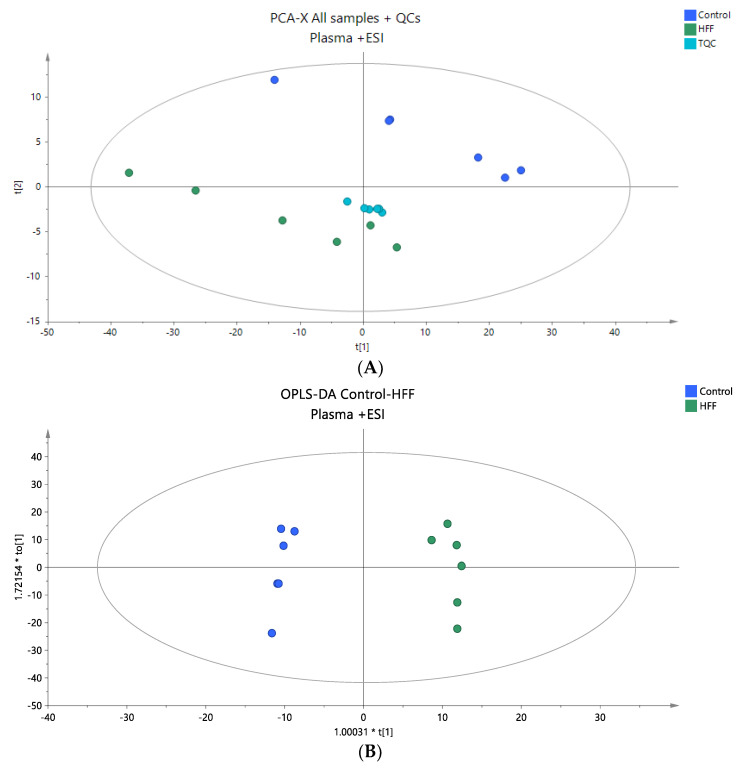
(**A**) A PCA score plot for the two studied groups, control and HFF, and QC samples were constructed for the analysis of plasma samples. QC samples are depicted in light blue and clustered together (R^2^X = 0.880, Q^2^ = 0.660). (**B**) The OPLS-DA plot illustrates the constructed model of control versus HFF (R^2^X = 0.786, R^2^Y = 0.989 Q^2^ = 0.867, CV ANOVA *p*-value 4.36 × 10^−3^). Logarithmic transformation of the data and Pareto scaling were used in all plasma models in positive ionization mode.

**Figure 2:**
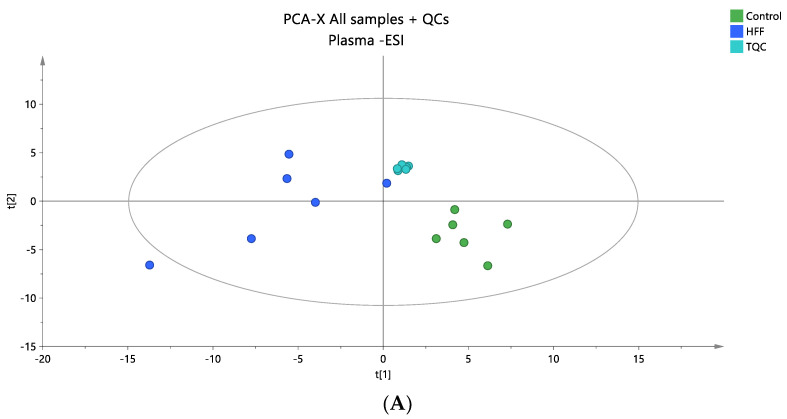

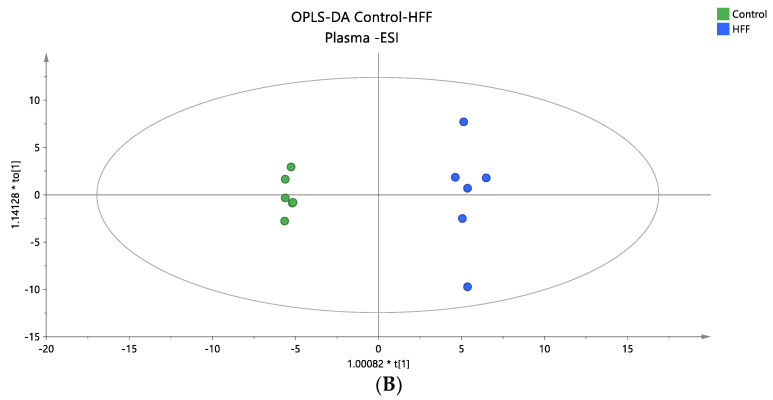
(**A**) A PCA score plot for the two studied groups, control and HFF, and QC samples were constructed for the analysis of plasma samples. QC samples are depicted in light blue and clustered together (R^2^X = 0.609, Q^2^ = 0.139). (**B**) The OPLS-DA plot illustrates the constructed model of control versus HFF (R^2^X = 0.419, R^2^Y = 0.994, Q^2^ = 0.835, CV ANOVA *p*-value 7.47 × 10^−3^). Logarithmic transformation of the data and Pareto scaling were used in all plasma models in negative ionization mode.

Untargeted lipidomic analysis was performed on the plasma samples of the rat model in positive and negative ionization modes to obtain the full information of the plasma lipidome of the studied groups. In total, 1676 ions were detected in positive mode after peak alignment resulting in 1187 signal ions after filtering, following the data processing methodology described in Section 2.6. In negative mode, 921 signal ions were detected, and 603 were considered for further statistical analysis after filtering (Section 2.5). The integrity and accuracy of the analytical data were first evaluated by examining quality control (QC) samples. The PCA score plots, encompassing all samples and QC samples, showed that the analytical precision was satisfactory, as the QCs were closely grouped together (Figure 1A and Figure 2A). Following the initial discrimination between control and HFF rats, as demonstrated in PCA plots (Figure 1A and Figure 2A), supervised OPLS-DA analysis was performed to find the statistically significant lipids (Figure 1B and Figure 2B). The quality metrics of the constructed models are presented in Table S2. In total, 37 lipid species (positive mode: 23, negative mode: 14)—among them, 5 sphingolipids, 12 phospholipids, 2 cholesterol esters, 1 diglyceride and 14 TG—were found to be dysregulated between the two groups; more specifically, they were decreased in the HFF group with the exception of PC 38:5, which was found to be elevated in the HFF group. Table 2 presents all information related to detailed annotations of the statistically significant lipid species revealed by this comparison. Additionally, Table 2 summarizes the statistical parameters of Log2FC, *p*-values, VIP, and CV% based on the comparison, indicating the impact on the lipid levels and their effect on the distinct profiles that were observed.

### 3.3. Lipidomic Profiling in Liver Tissue

**Figure 3:**
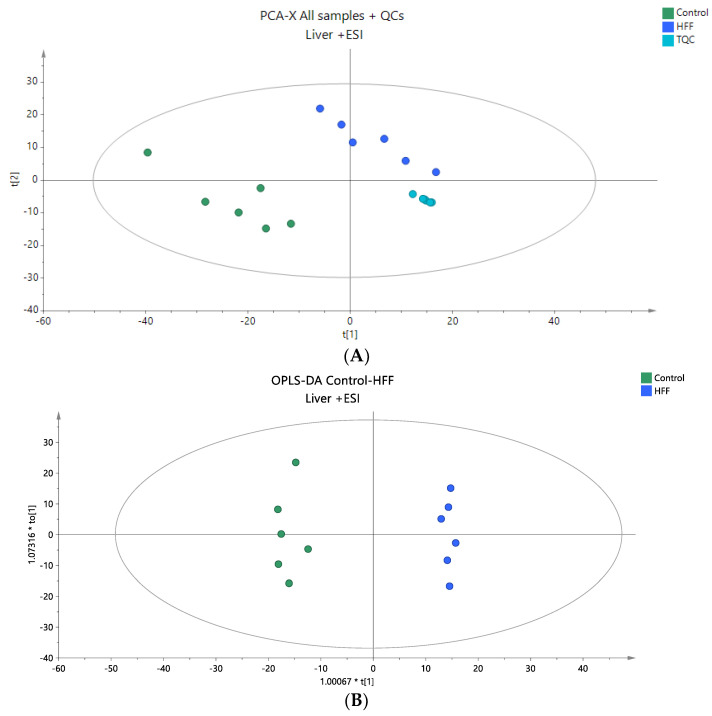
(**A**) A PCA score plot for two studied groups, control and HFF, and QC samples were constructed for the analysis of liver samples. QC samples are depicted in light blue and clustered together (R2X = 0.861, Q2 = 0.442). (**B**) The OPLS-DA plot illustrates the constructed model of control versus HFF (R2X = 0.602, R2Y = 0.986, Q2 = 0.949, CV ANOVA *p*-value 1.44 × 10^−4^). Logarithmic transformation of the data and Pareto scaling were used in all liver models in positive ionization mode.

**Figure 4:**
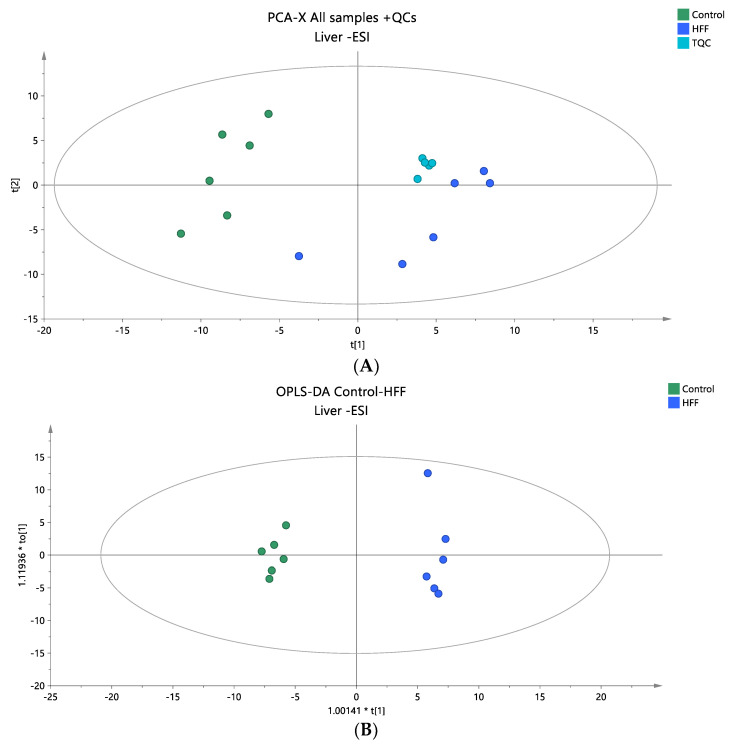
(**A**) A PCA score plot for two studied groups, control and HFF, and QC samples were constructed for the analysis of liver samples. QC samples are depicted in light blue and clustered together (R^2^X = 0.737, Q^2^ = 0.221). (**B**) The OPLS-DA plot illustrates the constructed model of control versus HFF (R^2^X = 0.519, R^2^Y = 0.991, Q^2^ = 0.896, CV ANOVA *p*-value 1.62 × 10^−3^). Logarithmic transformation of the data and Pareto scaling were used in all plasma models in negative ionization mode.

The liver tissue samples from the rat model were subjected to untargeted lipidomic analysis in both positive and negative ionization modes, aiming to comprehensively characterize the liver lipidome of the two groups. In the positive mode, peak alignment revealed 2671 ions, which were refined to 2171 signal ions after filtering, as detailed in Section 2.5. In the negative mode, 1223 signal ions were detected, with 610 retained for further analysis post filtering (Section 2.5). The integrity and accuracy of the analytical data were validated using QC samples. PCA score plots, incorporating all samples and QC samples, demonstrated sufficient analytical precision, as evidenced by the tight clustering of QC samples (Figure 3A and Figure 4A). Subsequently, a supervised OPLS-DA analysis was conducted to identify statistically significant lipids, as shown by the OPLS-DA plots (Figure 3B and Figure 4B). The quality metrics of the models constructed are provided in Table S2. A total of 43 lipid species were identified as dysregulated between the two groups, comprising 6 sphingolipids, 14 phospholipids, 1 cholesterol ester, 2 diglycerides, and 20 TG. Among these, all sphingolipids except Cer 40:2 O2 were decreased in the HFF group, all phospholipids were decreased in the HFF group except PC 38:4 and PC 40:5, and all glycerolipids were elevated in the HFF group. This information is summarized in Table 3, which includes detailed annotations of the statistically significant lipid species along with statistical parameters such as Log2FC, *p*-values, VIP, and CV%, indicating their impact on lipid levels and their contribution to the observed profiles.

### 3.4. Lipidomic Profiling in Adipose Tissue

**Figure 5:**
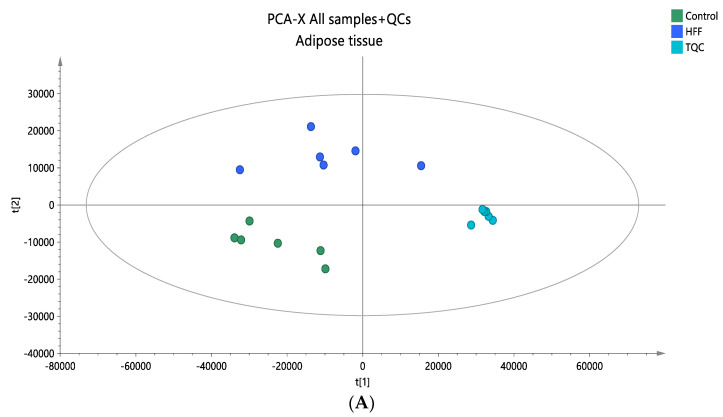

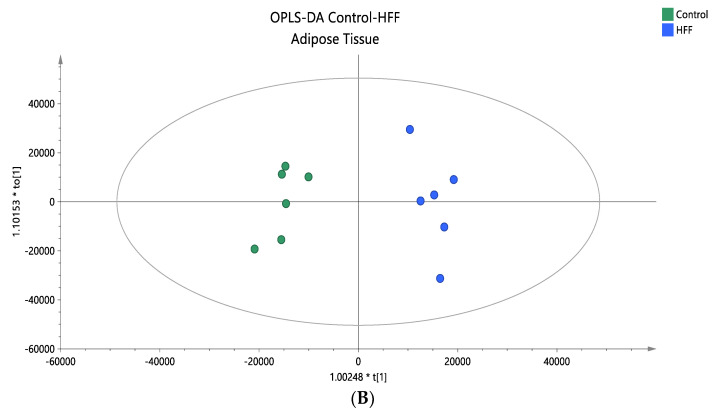
(**A**) A PCA score plot for the two studied groups, control and HFF, and QC samples were constructed. QC samples are depicted in light blue and clustered together (R^2^X = 0.886, Q^2^ = 0.676). (**B**) The OPLS-DA plot illustrates the constructed model of control versus HFF (R^2^X = 0.670, R^2^Y = 0.961, Q^2^ = 0.886, CV ANOVA *p*-value 2.00 × 10^−3^). Pareto scaling was used in all adipose tissue models.

A lipidomic analysis was carried out on the adipose tissue samples of the animal model using positive ionization mode to obtain comprehensive information on the adipose tissue lipidome. The analysis was performed only in positive mode, aiming to investigate the content of glycerolipids, which ionize more effectively under this condition. Following peak alignment, a total of 1703 ions were detected, leading to 1187 signal ions after filtering, as detailed in Section 2.5. The analytical data’s integrity and accuracy were first assessed by examining quality control (QC) samples. PCA score plots, which included all samples and QC samples, indicated that the analytical precision was adequate, as the QCs were closely grouped together (Figure 5A). A supervised OPLS-DA analysis was then performed to find statistically significant lipids, as demonstrated by the OPLS-DA plots (Figure 5B). The quality metrics of the constructed models are presented in Table S2. In total, 11 TG species were found to be dysregulated between the two groups, and more specifically, TG 48:2, TG 50:1, TG 50:2, and TG 50:3 were found elevated in HFF groups, while TG 54:3, TG 54:4, TG 54:5, TG 54:6, TG 56:3, TG 56:6, and TG 56:8 were found decreased in the HFF group. The observed trend in TG species, which showed statistical significance between the groups, could likely be attributed to the composition of saturated and monounsaturated fatty acids. This information is summarized in Table 4, which also includes detailed annotations of the statistically significant lipid species and statistical parameters such as Log2FC, *p*-values, VIP, and CV% that indicate the impact on lipid levels and their contribution to the observed profiles.

### 3.5. Common Lipid Species between Plasma, Liver and Adipose Tissue Samples

Our study revealed substantial alterations in lipid levels across a range of tissues, and several lipids displayed common changes. In the plasma and liver, 10 lipid species exhibited notable differences: SM 42:2;O2, SM 43:1;O2, LPC 17:0, PC 32:1, PC 33:2, PC 35:2, PC 38:6, TG 52:6, TG 53:3, and TG 58:9. These particular sphingomyelins and phospholipids were found to decrease in the HFF group in both plasma and liver samples, whereas TG species demonstrated disparate trends in plasma and liver. In the plasma, TGs were reduced, whereas they were elevated in the liver. In the plasma and adipose tissue, three TG species were common, namely TG 48:2, TG 50:3, and TG 56:6, and they were all decreased in both plasma and adipose tissue samples, except for TG 56:6, which was elevated only in the adipose tissue of the HFF group. Furthermore, the liver and adipose tissue exhibited changes in TG, with four species being common in both tissues: TG 54:3, TG 54:6, TG 56:3, and TG 56:8. All these species were elevated in the HFF group in both liver and adipose tissues. No statistically significant lipids were identified in all three tissues. The results of the common lipid levels across various tissues are presented in Table 5, which includes the Log2FC values for the common species in all matrices.

## 4. Discussion

In this study, HFF rats were affected by onset of dyslipidemia, as the predominant cholesterol fraction in rats, HDL-c, was significantly decreased (*p* < 0.05). Another unfavorable disorder induced by HFF feeding was elevated SBP (*p* < 0.05), a common alteration in MetS and a risk factor for cardiovascular disease [21]. Diets high in fats and fructose are well known to negatively alter blood pressure via several mechanisms, including increased salt retention, endothelial dysfunction, and overstimulation of the sympathetic nervous system [22].

Furthermore, we investigated lipidomics-based differentiation associated with MetS induced by a HFF diet in a rat model. Our aim was to address the question of how MetS affects the lipidome in key target biofluids and organs, including plasma, liver, and adipose tissue. We aimed to validate the relevant literature and ultimately propose potential biomarkers for this complex syndrome. Lipid profiling played a crucial role in analyzing specific lipid species to uncover their contribution to this complex syndrome [23].

The general picture that emerged from our analysis showed downregulation of several lipid species across the distinct lipid profiles of the analyzed matrices, with both unique and common lipid alterations observed in the three tissues. Using untargeted lipidomics, we identified a broad spectrum of lipid molecules, including glycerolipids, glycerophospholipids, sphingolipids, and cholesterol esters, as the primary lipid pools [24] from which these differences emerged.

Plasma lipid profiles showed a significant decrease in certain sphingolipids, phospholipids, and TG with 37 identified lipid species (determined by positive and negative ionization modes) being notably altered. Among these, PC 38:5 was the only lipid species that was increased in the HFF group. Interestingly, the observed decrease in TG in plasma contrasts with the accumulation of TG in the liver, revealing a dysregulation of lipid metabolism. This discrepancy could be due to increased lipid uptake by peripheral tissues, a known condition in MetS, and the accumulation of lipid droplets in the liver. In our previous study on an NAFLD rat model, we observed similar results in which hepatic accumulation of TG was associated with the pathophysiology of NAFLD, a metabolic condition closely related to MetS, and was attributed to the upregulation of de novo lipogenesis [15].

In addition to the accumulation of TG in the liver, other lipid species, including sphingolipids and phospholipids, were also dysregulated. A total of 43 lipid species were identified as dysregulated between the HFF and control groups, comprising 6 sphingolipids, 14 phospholipids, 1 cholesterol ester, 2 diglycerides, and 20 TG. Remarkably, all sphingolipids except Cer 40:2; O2 and all phospholipids except PC 38:4 and PC 40:5 were decreased in the HFF group, while all glycerolipids were elevated.

In adipose tissue, which is considered the main site of lipid storage [25], the observed statistically significant changes were also in contrast to hepatic TG accumulation. More specifically, adipose tissue showed a decrease in several TG species, probably caused by increased lipid mobilization from adipose tissue to non-adipose tissues, such as the liver. This finding is also related to the known condition of MetS, which is characterized by ectopic fat deposition [26,27,28]. As the adipose tissue is no longer able to cope with lipid overload, the liver takes over the role of a lipid sink under the pathological conditions of dyslipidemia and metabolic dysfunction [29,30,31,32]. This dysregulation of TG in adipose tissue was clearly observed in our findings, with 4 out of a total of 11 types being increased and 7 decreased. In a careful review of previous studies, rodent models [33,34,35,36,37,38,39,40,41,42,43,44,45], including Wistar and Sprague Dawley rats 46, were used in most cases to mimic conditions such as high-fat, high-glucose and high-fructose diets for the development of MetS. In these studies, a series of experimental designs were used to investigate the impact of dietary interventions and lifestyle factors on lipid metabolism. A successful rat model of MetS should simulate the conditions observed in patients, including obesity and increased adiposity, dyslipidemia (characterized by elevated TG and decreased HDL cholesterol), as well as altered lipid metabolism involving multiple lipid species, including phospholipids, sphingolipids, and cholesterol esters [46]. Characteristic features include impaired glucose tolerance and hyperinsulinemia leading to insulin resistance, which is also reflected in our results. Finally, hypertension and fatty liver possibly leading to non-alcoholic fatty liver disease (NAFLD) complete the picture of MetS.

As discussed in a related review [47], insulin resistance plays a fundamental role in the accumulation of lipids in adipocytes and non-adipose tissues such as the liver, leading to chronic inflammation caused by hypertrophy. In hypertrophied adipose tissue, neutral lipids accumulate in non-adipose tissues [47]. It appears that increased ceramide levels in these tissues have significant involvement in this process, further exacerbate insulin resistance in obese humans, rodents, and non-human primates fed on a Western diet. The high fructose content in a high-fat diet appears to worsen MetS enhancing by promoting de novo lipogenesis in the liver, leading to lipid accumulation that exacerbates insulin resistance and hepatic steatosis [48].

In agreement with our study, both Zhou et al. [39] and Lan et al. [35] observed significant changes in hepatic lipids induced by high-fat, high-sucrose, or high-fructose diets in rats. Zhou et al. [39] reported a progressive accumulation of free fatty acids (FFAs) and lipid droplets in the liver, leading to MetS and associated diseases such as NAFLD and type T2DM. Similarly, Lan et al. [35] observed changes in gene expression associated with increased production of saturated and monounsaturated fatty acids, leading to accumulation of TG in the liver.

In further support of our findings, Bacle et al. [33] and Tranchida et al. [34] observed alterations in phospholipid and fatty acid profiles, particularly shifts in monounsaturated and polyunsaturated lipid species in the liver, skeletal muscles, and plasma, in rodents subjected to high-fat high-fructose (HFF) diets. A significant decrease in polyunsaturated fatty acids and phospholipids with a concomitant increase in saturated fatty acids, consistent with our findings, was also reported by Gowda et al. [38] and Jové et al. [45]. In addition, Chan et al. [36] and Bacle et al. [33] observed inflammation and lipid rearrangements associated with adipose tissue dysfunction, which may potentially explain our observations of complex lipid mobilization and systemic dysregulation in MetS. The consistent elevation of PC 38:5 in plasma suggests its potential as a biomarker for MetS progression, requiring further validation to confirm its role.

Our study is limited by the relatively small number of rats per group. Another limitation arises from the lipid analyses, given that the complexity of tissue lipidome makes it difficult to comprehensively identify individual lipid species and understand their specific roles in the development of MetS.

Our findings provide strong evidence of lipid alterations associated with MetS, based on controlled animal model conditions, which may have implications for human clinical settings. Specific lipid dysregulations, such as changes in certain sphingolipids, phospholipids, and triglycerides observed in our study, could potentially serve as biomarkers for the early diagnosis and monitoring of MetS in patients, pending clinical validation in human cohorts. Furthermore, understanding the lipidomic profile changes in response to high-fat, high-fructose diets could guide dietary and lifestyle interventions aimed at regulating lipid metabolism, improving insulin sensitivity, and reducing cardiovascular risk in individuals at risk for MetS.

## 5. Conclusions

High-throughput lipidomic analysis of HFF-induced MetS in rats revealed marked alterations in the lipidomic profiles of plasma, liver and adipose tissue. Although some changes were tissue-specific, several sphingolipids, phosphatidilcholines, and triglycerides showed similar trends in all tissues. In most cases, lipids were downregulated in the HFF group. The exact mechanisms underlying these changes, as well as their potential clinical significance, require further investigations.

## Figures and Tables

**Table 1:** Physiological and biochemical parameters of controls and HFF-rats on a high-fat high-fructose (HFF) diet. The data are presented as mean values ± SD (*n* = 6). Parameters with statistically significant *p*-values < 0.05 are indicated with *, whereas parameters with a *p*-value < 0.001 are presented with ***.

	Control	HFF
Food intake (g/day/cage)	69.82 ± 6.14	47.52 ± 9.06 ***
Water/juice intake (mL/day/cage)	80.65 ± 8.43	69.90 ± 12.47
Calorie intake (kcal/day/rat)	79.98 ± 5.38	82.55 ± 6.21
Baseline body mass (g)	335 ± 26	347 ± 13
Final body mass (g)	365 ± 24	385 ± 16
Body fat (%)	3.24 ± 0.44	6.58 ± 1.21 ***
SBP (mmHg)	126 ± 9	148 ± 7 *
Total cholesterol (mmol/L)	1.54 ± 0.23	1.55 ± 0.12
HDL-c (mmol/L)	0.98 ± 0.18	0.80 ± 0.14 *
LDL-c (mmol/L)	0.23 ± 0.14	0.34 ± 0.17
TG (mmol/L)	0.77 ± 0.10	0.81 ± 0.19
Glc (mmol/L)	5.32 ± 0.26	5.60 ± 0.41

Abbreviations: SBP—systolic blood pressure, HDL-c—high-density lipoprotein cholesterol, LDL-c—low-density lipoprotein cholesterol, TG—triglycerides, Glc—glucose.

**Table 2:** Plasma lipid species found to be statistically significant between controls and HFF groups. Information is provided regarding the neutral formula and fatty acid chains of the lipids, molecular structure, monoisotopic masses of precursors, detected adducts, retention time, and mass accuracy. *p*-values, Log2FCs, CV% values, and VIP scores are also provided for each lipid after multivariate and univariate analysis. Student’s *t*-test and the Mann–Whitney U test were performed for normally and non-normally distributed species, respectively.

Bulk Number	Annotation	Neutral Formula	Exact Mass (*m*/*z*)	Exact Mass Adduct (*m*/*z*)	Monoisotopic Mass Adduct (*m*/*z*)	Δ ppm	Adduct	Rt (min)	*p*-Value	VIP	CV%	Log2FC
Cer 42:2;O2	Cer 42:2; O2	C42H81NO3	647.6216	692.6195	692.6198	−0.4	[M + HCOO]^−^	9.5	4.10 × 10 ^−3^	1.5	21.1	−1.0
SM 32:1;O2	SM 32:1; O2	C37H75N2O6P	674.5362	675.5433	675.5435	0.3	[M + H] ^+^	4.6	8.70 × 10 ^−3^	1.8	14.0	−2.9
SM 33:1;O2	SM 33:1; O2	C38H77N2O6P	688.5519	733.5497	733.5501	−0.5	[M + HCOO]^−^	5.2	6.66 × 10 ^−3^	1.2	2.5	−0.7
SM 41:1;O2	SM 41:1; O2	C46H93N2O6P	800.6771	835.6465	835.6465	0.0	[M + Cl]^−^	9.1	1.09 × 10 ^−3^	1.4	11.4	−0.9
SM 42:2;O2	SM 42:2; O2	C47H93N2O6P	812.6771	813.6883	813.6844	−4.8	[M + H] ^+^	8.8	4.30 × 10 ^−3^	1.6	11.0	−2.6
SM 43:1;O2	SM 43:1; O2	C48H97N2O6P	828.7084	873.7090	873.7066	2.7	[M + HCOO]^−^	9.5	6.23 × 10 ^−4^	1.8	4.1	−1.4
LPC 17:0	LPC 17:0	C25H52NO7P	509.3481	554.3411	554.3463	−9.4	[M + HCOO]^−^	1.5	4.42 × 10 ^−4^	1.6	8.1	−1.1
LPC 22:6	LPC 22:6	C30H50NO7P	567.3325	612.3250	612.3307	−9.3	[M + HCOO]^−^	1.0	2.04 × 10 ^−3^	1.6	11.4	−1.1
PC 31:0	PC 15:0_16:0	C39H78NO8P	719.5465	720.5546	720.5538	−1.1	[M + H] ^+^	6.8	4.30 × 10 ^−3^	1.8	12.9	−3.0
PC 32:1	PC 16:0_16:1 PC 14:0_18:1	C40H78NO8P	731.5465	776.5444	776.5447	−0.4	[M + HCOO]^−^	6.2	5.15 × 10 ^−4^	1.9	3.0	−1.5
PC 33:1	PC 15:0_18:1	C41H80NO8P	745.5621	746.5700	746.5694	−0.8	[M + H] ^+^	7.0	6.10 × 10 ^−3^	1.6	11.3	−2.4
PC 33:2	PC 15:0_18:2	C41H78NO8P	743.5465	788.5448	788.5447	0.1	[M + HCOO]^−^	5.8	4.30 × 10 ^−3^	1.4	4.5	−1.0
PC 35:2	PC 17:0_18:2	C43H82NO8P	771.5778	816.5763	816.5760	0.4	[M + HCOO]^−^	7.4	2.28 × 10 ^−4^	1.6	5.8	−1.1
PC 36:5	PC 16:1_20:4	C44H78NO8P	779.5465	824.5381	824.5447	−8.0	[M + HCOO]^−^	5.1	2.24 × 10 ^−3^	1.9	20.9	−1.3
PC 37:4	PC 17:0_20:4	C45H82NO8P	795.5778	796.5883	796.5851	−4.1	[M + H] ^+^	7.1	4.30 × 10 ^−3^	1.6	9.6	−2.4
PC 38:5	PC 16:0_22:5 PC 18:0_20:5	C46H82NO8P	807.5778	852.5776	852.5760	1.9	[M + HCOO]^−^	7.0	2.20 × 10 ^−3^	1.9	16.5	1.9
PC 38:6	PC 16:0_22:6	C46H80NO8P	805.5622	850.5613	850.5604	1.1	[M + HCOO]^−^	5.9	3.56 × 10 ^−6^	1.7	20.0	−1.2
PC 40:7	PC 18:1_22:6	C48H82NO8P	831.5778	876.5784	876.5760	2.7	[M + HCOO]^−^	6.1	3.96 × 10 ^−4^	2.2	3.3	−1.7
PE 36:4	PE 16:0_20:4	C41H74NO8P	739.5152	738.5087	738.5079	1.1	[M-H]^−^	6.8	2.08 × 10 ^−2^	1.3	4.5	−0.8
CE 16:1	CE 16:1	C43H74O2	622.5688	640.6017	640.6027	1.5	[M + NH4] ^+^	12.2	1.52 × 10-^2^	1.9	11.0	−2.9
CE 20:4	CE 20:4	C47H76O2	672.5845	690.6206	690.6183	−3.3	[M + NH4] ^+^	11.9	3.81 × 10 ^−2^	1.8	20.7	−3.1
DG 40:8	DG 18:2_22:6	C43H68O5	664.5066	682.5396	682.5405	1.4	[M + H] ^+^	7.9	1.90 × 10 ^−2^	2.4	15.2	−7.4
TG 48:2	TG 14:0_16:0_18:2 TG 14:0_16:1_18:1 TG 16:0_16:1_16:1	C51H94O6	802.7050	820.7392	820.7389	−0.3	[M + NH4]^+^	11.6	4.30 × 10 ^−3^	2.0	17.3	−3.2
TG 49:2	TG 15:0_16:0_18:2	C52H96O6	816.7207	834.7547	834.7545	−0.3	[M + NH4]^+^	11.8	4.30 × 10 ^−3^	2.1	13.8	−3.6
TG 50:3	TG 14:0_18:1_18:2 TG 16:0_16:1_18:2 TG 16:1_16:1_18:1	C53H96O6	828.7207	846.7575	846.7545	−3.5	[M + NH4]^+^	11.6	8.70 × 10 ^−3^	1.6	10.3	−2.3
TG 51:2	TG 15:0_18:0_18:2 TG 15:0_18:1_18:1 TG 16:0_17:0_18:2	C54H100O6	844.7520	862.7866	862.7868	0.2	[M + NH4]^+^	12.3	8.70 × 10 ^−3^	2.2	24.5	−3.6
TG 51:3	TG 15:0_18:1_18:2 TG 16:0_17:1_18:2 TG 17:1_17:1_17:1	C54H98O6	842.7363	860.7717	860.7702	−1.7	[M + NH4]^+^	11.8	8.70 × 10 ^−3^	1.6	10.8	−2.3
TG 51:4	TG 15:0_18:2_18:2	C54H96O6	840.7207	858.7562	858.7545	−2.0	[M + NH4]^+^	11.4	4.11 × 10 ^−2^	1.7	15.1	−2.1
TG 52:5	TG 16:0_18:2_18:3 TG 16:1_18:1_18:3 TG 16:1_18:2_18:2	C55H96O6	852.7207	870.7588	870.7545	−4.9	[M + NH4]^+^	11.3	1.52 × 10 ^−2^	1.6	18.3	−2.4
TG 52:6	TG 16:0_16:2_20:4 TG 16:0_18:2_18:4 TG 16:0_18:3_18:3 TG 16:1_16:1_20:4 TG 16:1_18:1_18:4 TG 16:1_18:2_18:3 TG 16:2_18:1_18:3 TG 16:2_18:2_18:2	C55H94O6	850.7050	868.7404	868.7389	−1.7	[M + NH4]^+^	11.0	1.52 × 10 ^−2^	1.9	19.6	−3.1
TG 53:3	TG 16:0_18:2_19:1 TG 17:0_18:1_18:2	C56H102O6	870.7676	888.8034	888.8015	−2.1	[M + NH4]^+^	12.3	1.52 × 10 ^−2^	2.0	21.5	−2.8
TG 54:1	TG 16:0_16:0_22:1 TG 16:0_18:0_20:1 TG 16:0_18:1_20:0 TG 18:0_18:0_18:1	C57H108O6	888.8146	906.8474	906.8484	1.1	[M + NH4]^+^	13.0	2.60 × 10 ^−2^	2.0	25.3	−3.6
TG 56:6	TG 16:0_18:1_22:5 TG 16:0_18:2_22:4 TG 18:0_18:2_20:4 TG 18:1_18:1_20:4 TG 18:1_18:2_20:3 TG 18:2_18:2_20:2	C59H102O6	906.7676	924.8013	924.8015	0.2	[M + NH4]^+^	11.0-12.5	2.20 × 10 ^−3^	2.0	5.9	−4.1
TG 56:9	TG 18:2_18:2_20:5 TG 18:2_18:3_20:4	C59H96O6	900.7207	918.7586	918.7545	−4.4	[M + NH4]^+^	10.7	4.30 × 10 ^−3^	1.7	11.9	−3.1
TG 58:9	TG 18:1_20:4_20:4 TG 18:2_18:2_22:5 TG 18:2_20:3_20:4	C61H100O6	928.7520	946.7817	946.7858	4.3	[M + NH4]^+^	10.9	8.70 × 10 ^−3^	1.7	11.5	−3.3
TG 58:9	TG 18:1_18:2_22:6 TG 18:1_20:4_20:4 TG 18:2_18:2_22:5	C61H100O6	928.7520	946.7905	946.7858	−4.9	[M + NH4]^+^	11.3	8.70 × 10 ^−3^	1.6	19.3	−2.9
TG 60:9	TG 18:1_20:3_22:5 TG 18:1_20:4_22:4 TG 20:3_20:3_20:3	C63H104O6	956.7832	974.81868	974.8171	−1.6	[M + NH4]^+^	11.3	3.81 × 10 ^−2^	2.0	24.5	−4.2

**Table 3:** Liver lipid species found to be statistically significant between the control and HFF groups. Information is provided regarding the neutral formula and fatty acid chains of the lipids, molecular structure, monoisotopic masses of precursors, detected adducts, retention time, and mass accuracy. *p*-values, Log2FCs, CV% values, and VIP scores are also provided for each lipid after multivariate and univariate analysis. Student’s *t*-test and the Mann–Whitney U test were performed for normally and non-normally distributed species, respectively.

Bulk Number	Annotation	Neutral Formula	Exact Mass (*m*/*z*)	Exact Mass Adduct (*m*/*z*)	Monoisotopic Mass Adduct (*m*/*z*)	Δ ppm	Adduct	Rt (min)	*p*-Value	VIP	CV%	Log2FC
Cer 38:1;O2	Cer 38:1; O2	C38H75NO3	593.5747	638.5732	638.5729	−0.5	[M + HCOO]^−^	8.9	4.32 × 10 ^−4^	1.5	7.6	−1.4
Cer 40:2;O2	Cer 40:2; O2	C40H77NO3	619.5903	664.5885	664.5885	0.0	[M + HCOO]^−^	8.9	4.92 × 10 ^−4^	1.4	9.8	1.2
Cer 41:2;O2	Cer 41:2; O2	C41H79NO3	633.6060	678.6028	678.6042	2.1	[M + HCOO]^−^	9.3	1.06 × 10 ^−2^	1.2	9.6	−1.0
Cer 43:1;O2	Cer 43:1; O2	C43H85NO3	663.6530	708.6525	708.6511	−2.0	[M + HCOO]^−^	10.2	3.64 × 10 ^−4^	1.2	16.1	−0.9
SM 42:2;O2	SM 42:2; O2	C47H93N2O6P	812.6771	857.6765	857.6753	−1.4	[M + HCOO]^−^	8.8	1.88 × 10 ^−3^	1.3	24.8	−1.1
SM 43:1;O2	SM 43:1; O2	C48H97N2O6P	828.7084	873.7081	873.7066	−1.7	[M + HCOO]^−^	9.5	4.17 × 10 ^−5^	1.3	12.6	−1.0
LPC 17:0	LPC 17:0	C25H52NO7P	509.3481	554.3463	554.3463	0.0	[M + HCOO]^−^	1.5	3.62 × 10 ^−4^	1.4	16.8	−1.2
PC 32:1	PC 16:0_16:1	C40H78NO8P	731.5465	776.5454	776.5447	−0.9	[M + HCOO]^−^	6.1	2.98 × 10 ^−6^	1.5	14.7	−1.4
PC 33:2	PC 15:0_18:2	C41H78NO8P	743.5465	788.5442	788.5447	0.6	[M + HCOO]^−^	5.8	2.81 × 10 ^−2^	1.3	11.7	−0.9
PC 35:2	PC 17:0_18:2	C43H82NO8P	771.5778	816.5764	816.5760	−0.5	[M + HCOO]^−^	7.4	9.79 × 10 ^−5^	1.4	11.2	−1.1
PC 35:4	PC 15:0_20:4	C43H78NO8P	767.5465	812.5459	812.5447	−1.5	[M + HCOO]^−^	5.5	1.68 × 10 ^−4^	1.2	8.0	−0.9
PC 36:1	PC 18:0_18:1	C44H86NO8P	787.6091	832.6092	832.6073	−2.3	[M + HCOO]^−^	8.4	1.54 × 10 ^−3^	1.2	19.4	−0.9
PC 38:4	PC 16:0_22:4	C46H84NO8P	809.5935	854.5933	854.5917	−1.9	[M + HCOO]^−^	7.5	4.03 × 10 ^−3^	2.2	10.3	3.1
PC 38:6	PC 16:0_22:6	C46H80NO8P	805.5622	850.5606	850.5604	−0.2	[M + HCOO]^−^	5.8	1.90 × 10 ^−2^	1.2	23.9	−1.0
PC 40:5	PC 18:0_22:5	C48H86NO8P	835.6091	880.6100	880.6073	−3.1	[M + HCOO]^−^	8.2	4.30 × 10 ^−3^	3.1	10.6	6.5
PE 37:4	PE 17:0_20:4	C42H76NO8P	753.5309	752.5243	752.5236	−0.9	[M−H]^−^	7.6	2.20 × 10 ^−3^	1.2	14.0	−1.1
PE O−36:5	PE *p*-16:0_20:4	C41H74NO7P	723.5202	722.5130	722.5130	0.0	[M−H]^−^	7.5	9.96 × 10 ^−3^	1.2	18.6	−1.1
PG 40:8	PG 18:2_22:6	C46H75O10P	818.5097	817.5022	817.5025	0.4	[M−H]^−^	3.6	2.20 × 10 ^−3^	1.5	25.2	−1.6
PI 37:4	PI 17:0_20:4	C46H81O13P	872.5415	871.5360	871.5342	−2.1	[M−H]^−^	5.3	1.52 × 10 ^−2^	2.0	24.6	−2.5
PI 40:6	PI 18:0_22:6	C49H83O13P	910.5571	909.5507	909.5499	−0.9	[M−H]^−^	5.6	2.20 × 10 ^−3^	1.5	18.4	−1.8
CE 18:1	CE 18:1	C45H78O2	650.6001	668.6351	668.6340	1.7	[M + NH4] ^+^	12.8	3.24 × 10 ^−4^	2.3	7.7	1.5
DG 40:3	DG 18:0_22:3	C43H78O5	674.5849	692.6180	692.6187	−0.9	[M + NH4] ^+^	9.0	3.94 × 10 ^−3^	2.3	6.7	1.4
DG 40:5	DG18:1_22:4	C43H74O5	670.5536	688.5886	688.5874	1.7	[M + NH4] ^+^	8.7	7.22 × 10 ^−3^	2.2	4.6	1.6
TG 50:5	TG 14:0_18:2_18:3 TG 14:1_18:1_18:3 TG 14:1_18:2_18:2 TG 14:2_18:1_18:2 TG 16:1_16:1_18:3	C53H92O6	824.6894	842.7250	842.7232	2.2	[M + NH4] ^+^	10.8	2.35 × 10 ^−2^	2.6	5.7	1.6
TG 52:6	TG 16:0_18:3_18:3 TG 16:1_18:2_18:3	C55H94O6	850.7050	868.7432	868.7389	5.0	[M + NH4] ^+^	10.8	4.16 × 10 ^−2^	2.1	24.7	1.4
TG 53:2	TG 16:0_18:1_19:1 TG 16:0_18:2_19:0 TG 17:0_18:0_18:2 TG 17:0_18:1_18:1	C56H104O6	872.7833	890.8182	890.8171	1.2	[M + NH4] ^+^	12.8	8.25 × 10 ^−4^	2.4	8.1	1.3
TG 53:3	TG 16:0_18:2_19:1 TG 16:1_18:1_19:1 TG 17:0_18:1_18:2 TG 17:1_18:0_18:2 TG 17:1_18:1_18:1	C56H102O6	870.7676	888.8018	888.8015	0.3	[M + NH4] ^+^	12.3	2.07 × 10 ^−3^	2.1	4.5	1.2
TG 53:4	TG 17:0_18:2_18:2 TG 17:1_18:1_18:2 TG 17:2_18:1_18:1	C56H100O6	868.7520	886.7856	886.7858	−0.2	[M + NH4] ^+^	11.9	6.72 × 10 ^−3^	2.2	4.8	1.3
TG 53:5	TG 15:0_18:1_20:4 TG 15:0_18:2_20:3 TG 17:0_18:2_18:3 TG 17:1_18:1_18:3 TG 17:1_18:2_18:2 TG 17:2_18:1_18:2	C56H98O6	866.7363	884.7726	884.7702	2.8	[M + NH4] ^+^	11.4	3.20 × 10 ^−2^	2.3	8.1	1.4
TG 54:2	TG 16:0_18:1_20:1 TG 18:0_18:1_18:1	C57H106O6	886.7989	904.8384	904.8328	6.2	[M + NH4] ^+^	13.0	1.07 × 10 ^−3^	2.4	4.6	1.6
TG 54:3	TG 16:0_18:1_20:2 TG 16:0_18:2_20:1 TG 18:0_18:1_18:2 TG 18:1_18:1_18:1	C57H104O6	884.7833	902.8201	902.8171	3.3	[M + NH4] ^+^	12.6	1.13 × 10 ^−3^	2.2	6.0	1.4
TG 54:6	TG 18:1_18:2_18:3 TG 18:2_18:2_18:2	C57H98O6	878.7363	896.7761	896.7702	6.6	[M + NH4] ^+^	11.2	1.08 × 10 ^−3^	2.3	3.5	1.5
TG 54:8	TG 18:2_18:3_18:3	C57H94O6	874.7050	892.7427	892.7389	4.2	[M + NH4] ^+^	10.6	2.50 × 10 ^−3^	2.3	7.0	1.2
TG 56:3	TG 16:0_20:1_20:2 TG 18:0_18:1_20:2 TG 18:0_18:2_20:1 TG 18:1_18:1_20:1 TG 18:1_18:2_20:0	C59H108O6	912.8146	930.8547	930.8484	6.8	[M + NH4] ^+^	13.0	6.44 × 10 ^−3^	2.8	27.7	2.3
TG 56:4	TG 16:0_18:1_22:3 TG 16:0_20:1_20:3 TG 18:0_18:1_20:3 TG 18:0_18:2_20:2 TG 18:0_18:3_20:1 TG 18:1_18:1_20:2 TG 18:1_18:2_20:1	C59H106O6	910.7989	928.8389	928.8328	6.6	[M + NH4] ^+^	12.6	1.09 × 10 ^−3^	2.6	4.5	1.8
TG 56:8	TG 18:2_18:2_20:4	C59H98O6	902.7363	920.7751	920.7702	5.3	[M + NH4] ^+^	11.1	1.50 × 10 ^−3^	2.1	6.2	1.3
TG 58:6	TG 16:0_20:1_22:5 TG 16:0_20:2_22:4 TG 18:0_18:1_22:5 TG 18:0_18:2_22:4 TG 18:0_20:2_20:4 TG 18:1_18:1_22:4 TG 18:1_18:2_22:3 TG 18:1_20:1_20:4 TG 18:1_20:2_20:3 TG 18:2_20:1_20:3 TG 18:2_20:2_20:2	C61H106O6	934.7989	952.8382	952.8328	5.7	[M + NH4] ^+^	12.3	5.00 × 10 ^−3^	2.9	4.4	2.4
TG 58:7	TG 16:0_20:2_22:5 TG 18:0_18:2_22:5 TG 18:1_18:1_22:5 TG 18:1_18:2_22:4 TG 18:1_20:2_20:4 TG 16:0_20:3_22:4 TG 18:2_18:2_22:3 TG 18:0_20:3_20:4 TG 18:1_18:3_22:3	C61H104O6	932.7833	950.8228	950.8171	6.0	[M + NH4] ^+^	11.9	1.82 × 10 ^−3^	2.4	13.7	1.6
TG 58:8	TG 18:1_18:2_22:5 TG 18:1_18:3_22:4 TG 18:1_20:3_20:4 TG 18:2_18:2_22:4 TG 18:2_20:3_20:3	C61H102O6	930.7676	948.8078	948.8015	6.7	[M + NH4] ^+^	11.4	1.09 × 10 ^−3^	2.2	6.2	1.4
TG 58:9	TG 18:1_20:4_20:4 TG 18:2_20:3_20:4	C61H100O6	928.7520	946.7876	946.7858	1.9	[M + NH4] ^+^	10.7	1.84 × 10 ^−3^	2.2	17.8	1.3
TG 60:7	TG 18:1_20:2_22:4 TG 16:0_22:3_22:4	C63H108O6	960.8145	978.8505	978.8484	2.2	[M + NH4] ^+^	11.9	1.37 × 10 ^−3^	2.8	3.0	2.0
TG 60:8	TG 18:1_20:1_22:6 TG 18:1_20:2_22:5 TG 18:2_20:1_22:5	C63H106O6	958.7989	976.8374	976.8328	4.7	[M + NH4] ^+^	11.4	9.60 × 10 ^−3^	2.7	7.2	2.2
TG 60:10	TG 18:2_20:3_22:5 TG 18:2_20:4_22:4	C63H102O6	954.7676	972.8066	972.8015	5.2	[M + NH4] ^+^	11.3	7.15 × 10 ^−3^	2.9	4.6	2.0

**Table 4:** Adipose tissue lipid species found to be statistically significant between control and HFF groups. Information is provided regarding the neutral formula and fatty acid chains of the lipids, molecular structure, monoisotopic masses of precursors, detected adducts, retention time, and mass accuracy. *p*-values, Log2FCs, CV% values, and VIP scores are also provided for each lipid after multivariate and univariate analysis. Student’s *t*-test and the Mann–Whitney U test were performed for normally and non-normally distributed species, respectively.

Bulk Number	Annotation	Neutral Formula	Exact Mass	Exact Mass Adduct *(m/z)*	Monoisotopic Mass Adduct *(m/z)*	Δ ppm	Adduct	Rt (min)	*p* Value Students’ Test	VIP	CV%	Log2FC
TG 48:2	TG 12:0_18:1_18:1 TG 14:0_16:0_18:2 TG 14:0_16:1_18:1 TG 16:0_16:1_16:1	C51H94O6	802.7050	820.7389	820.7389	0.0	[M + NH4] ^+^	11.7	4.36 × 10 ^−2^	2.3	2.7	−0.6
TG 50:1	TG 16:0_16:0_18:1	C53H100O6	832.7520	850.7785	850.7858	8.6	[M + NH4] ^+^	12.7	4.93 × 10 ^−2^	4.3	1.0	−0.6
TG 50:2	TG 14:0_18:1_18:1 TG 16:0_16:0_18:2	C53H98O6	830.7363	848.7699	848.7702	0.4	[M + NH4] ^+^	12.2	2.48 × 10 ^−3^	6.2	1.0	−0.9
TG 50:3	TG 14:0_18:1_18:2 TG 16:0_16:1_18:2 TG 16:1_16:1_18:1	C53H96O6	828.7207	846.7560	846.7545	−1.8	[M + NH4] ^+^	11.7	4.18 × 10 ^−2^	2.8	2.3	−0.6
TG 54:3	TG 16:0_18:2_20:1 TG 18:1_18:1_18:1	C57H104O6	884.7833	902.8172	902.8171	−0.1	[M + NH4] ^+^	12.8	3.18 × 10 ^−3^	9.1	1.0	1.3
TG 54:4	TG 18:0_18:1_18:3 TG 18:0_18:2_18:2 TG 18:1_18:1_18:2	C57H102O6	882.7676	900.8076	900.8015	−6.8	[M + NH4] ^+^	12.3	3.94 × 10 ^−4^	9.9	1.3	1.0
TG 54:5	TG 18:1_18:1_18:3 TG 18:1_18:2_18:2	C57H100O6	880.7520	898.7858	898.7858	0.0	[M + NH4] ^+^	11.8	6.39 × 10 ^−4^	8.7	0.9	1.1
TG 54:6	TG 18:1_18:2_18:3 TG 18:2_18:2_18:2	C57H98O6	878.7363	896.7701	896.7702	0.1	[M + NH4] ^+^	11.4	1.39 × 10 ^−3^	9.1	27.4	1.2
TG 56:3	TG 18:0_18:1_20:2 TG 18:0_18:2_20:1 TG 18:1_18:1_20:1 TG 18:1_18:2_20:0	C59H108O6	912.8146	930.8471	930.8484	1.4	[M + NH4] ^+^	12.8	8.09 × 10 ^−4^	2.3	0.9	0.7
TG 56:6	TG 16:0_18:2_22:4 TG 18:2_18:2_20:2	C59H102O6	906.7676	924.7969	924.8015	5.0	[M + NH4] ^+^	11.4	1.78 × 10 ^−4^	2.4	1.8	0.7
TG 56:8	TG 18:2_18:2_20:4	C59H98O6	902.7363	920.7657	920.7702	4.9	[M + NH4] ^+^	11.2	1.92 × 10 ^−2^	1.7	2.9	1.0

**Table 5:** Log2FC values of the common statistically significant lipids across the different matrices.

Plasma–Liver			Plasma–Adipose tissue			Liver–Adipose Tissue		
Lipids	Log2FC Plasma	Log2FC Liver	Lipids	Log2FC Plasma	Log2FC Adipose tissue	Lipids	Log2FC Liver	Log2FC Adipose Tissue
SM 42:2;O2	−2.6	−1.1	TG 48:2	−3.2	−0.6	TG 54:3	1.4	1.3
SM 43:1;O2	−1.4	−1.0	TG 50:3	−2.3	−0.6	TG 54:6	1.5	1.2
LPC 17:0	−1.1	−1.2	TG 56:6	−4.1	0.7	TG 56:3	2.3	0.7
PC 32:1	−1.5	−1.4				TG 56:8	1.3	1.0
PC 33:2	−1.0	−0.9						
PC 35:2	−1.1	−1.1						
PC 38:6	−1.2	−1.0						
TG 52:6	−3.1	1.4						
TG 53:3	−2.8	1.2						
TG 58:9	−3.3	1.3						

## Data Availability

Data are available upon reasonable request to the corresponding author.

## Supplementary Materials

The following supporting information can be downloaded at: https://www.mdpi.com/article/10.3390/nu16203466/s1, Table S1: Description of the extractions performed on the rat liver and adipose tissue samples; Table S2: Characteristics of the constructed unsupervised and supervised models. Logarithmic transformation of the data and Pareto scaling were used in all models in plasma and liver tissue, while only Pareto scaling was used in adipose tissue
