# Supplementary material for: Unveiling Lipidomic Alterations in Metabolic Syndrome: A Study of Plasma, Liver, and Adipose Tissues in a Dietary-Induced Rat Model
Source: Nutrients. 2024 Oct 13;16(20):3466. doi: 10.3390/nu16203466 (PMC11509917; doi:10.3390/nu16203466)
Supplement: Supplementary file 1 [file nutrients-16-03466-s001.zip › nutrients-3253198-supplementary.pdf]

Supplementary Table S1. Description of the extractions performed on the mice liver and adipose tissue samples.

		Liver Extraction		Adipose tissue (AT) Extraction				
	Mice_ID	mg Liver	MTBE: MeOH (3:1 v/v) (µL)	mg AT	MeOH (µL)	$CHCl^3$:MeOH (7:1 v/v) (µL)	$CHCl^3$ (µL)	H_2O (µL)
1	Control_1	31.2	1102	11.6	182	583	328	164
2	Control_2	29.0	1025	11.6	183	584	329	164
3	Control_3	29.3	1035	11.0	173	555	312	156
4	Control_4	32.1	1134	10.9	171	546	307	154
5	Control_5	28.8	1018	9.53	150	479	270	135
6	Control_6	28.9	1020	11.9	187	599	337	169
7	HFF_1	31.4	1108	10.7	168	536	302	151
8	HFF_2	30.3	1072	9.80	154	493	277	139
9	HFF_3	30.9	1093	8.68	136	436	245	123
10	HFF_4	31.1	1098	10.1	159	508	286	143
11	HFF_5	27.6	975	9.24	145	465	261	131
12	HFF_6	30.5	1077	12.7	200	640	360	180

Supplementary Table S2. Characteristics of the constructed unsupervised and supervised models. Logarithmic transformation of the data and pareto scaling were used in all models in plasma and liver tissue, while only pareto scaling was used in adipose tissue.

**Models**	**Type**	**N**	**R^2X**	**R^2Y**	**Q^2**	**CV ANOVA**
		Serum +ESI				
Control-HFF-QCs	PCA-X	18	0.880		0.660	
Control-HFF	PCA-X	12	0.874		0.650	
Control-HFF	OPLS-DA	12	0.786	0.989	0.867	4.36×10^{-3}
		Serum -ESI				
Control-HFF-QCs	PCA-X	18	0.609		0.139	
Control-HFF	PCA-X	12	0.453		0.127	
Control-HFF	OPLS-DA	12	0.419	0.994	0.835	7.47×10^{-3}
		Liver +ESI				
Control-HFF-QCs	PCA-X	18	0.861		0.442	
Control-HFF	PCA-X	12	0.901		0.231	
Control-HFF	OPLS-DA	12	0.602	0.986	0.949	1.44×10^{-4}
		Liver -ESI				
Control-HFF-QCs	PCA-X	17	0.737		0.221	
Control-HFF	PCA-X	12	0.714		0.221	
Control-HFF	OPLS-DA	12	0.519	0.991	0.896	1.62×10^{-3}
		Adipose tissue +ESI				
Control-HFF-QCs	PCA-X	18	0.886		0.676	
Control-HFF	PCA-X	12	0.933		0.416	
Control-HFF	OPLS-DA	12	0.670	0.961	0.886	2.00×10^{-3}
